# Hospital and Institutional News

**Published:** 1920-07-17

**Authors:** 


### HOSPITAL AND INSTITUTIONAL NEWS.

### SIR CLIFFORD ALLBUTT.

WE learn with pleasure that Sir Thomas Clifford Allbutt is to be sworn a member of the Privy Council. The greatness of the honour so conferred will bring considerable satisfaction to the hospital world, the more so when it is realised how few of the present Privy Councillors have become such from that most honourable reason, purely scientific attainment. With our contemporaries we believe that no medical man has ever before attained to this great distinction in recognition of pathological and professional work.

### EPSOM COLLEGE.

IN common with so many "medical" institutions, Epsom College has been passing through a period of financial difficulties which have caused no little anxiety to its promoters. The annual report shows a deficit of £4,425 incurred during the year 1919. This is largely accounted for by the nowadays customary needs for extensive repairs which accumulated during the war, and also by the generally increased cost of running the establishment. The main effect of this will be an increase in the school fees payable from September next. Sons of medical men will then have to pay 100 guineas, others 120 guineas, and day boys forty guineas, as compared with the former charges of seventy, eighty, and twenty-five guineas respectively. We read also that the sum of £6,000 will shortly have to be repaid to the mortgagees, while interest on the remaining £8,000 of the mortgage must be raised from 4½ to 5 per cent. In view, therefore, of this situation, the Council has decided to create mortgage debenture bonds of £100 each on the security of the college lands and buildings, in order to pay off both mortgage and bankers' loan. Friends of this well-known college are now asked to take up these bonds, with the double purpose of assisting in the provision of a public-school education for the sons of medical men at reduced fees, and of affording financial support to necessitous doctors, their widows, and families. It is an aim which should commend itself to all members of the medical profession.

### A SURGICAL CONGRESS.

NEXT week will be held, in Paris, an important International Congress on Surgery. One of the most interesting sections to come up for special consideration is that dealing with the surgical procedures possible in connection with the heart and blood-vessels. Certain possibilities in this work are already well known, such as removal of foreign bodies embedded in the cardiac musculature, massage of the heart following cases of sudden "death," as from artificial anæsthesia, etc., and the various methods of dealing surgically with aneurysmal lesions, but without doubt there will be many new developments brought to more general notice. The effects of radium or $x$-rays on new growths form a subject upon which European surgeons have been relatively reticent, but at this meeting this section receives particular attention. Similarly the developments in treatment of compound fractures and wound infections will be reviewed in the light of war experiences now applied to civil work for a year or more. We look forward with great interest to the invaluable discussions which will be heard.

### HOSPITALS AND TRADE UNIONS.

THE hospitals in this country have not yet had much to do with the trades unions as regards the side of their work which entails the employment of unskilled or semi-skilled persons, such as stokers, porters, laboratory attendants, charwomen, and the like. Recently the Dock, Wharf, Riverside, and General Worker's Union has attempted to organise this labour and several London hospitals—namely, the London, St. Mary's, Royal Free, Great Northern Central, King's College, Metropolitan, and Royal Waterloo hospitals, agreed to the arbitration of an Industrial Court, whose decision, and the proceedings upon which it was based, is detailed at length on page 409. Mr. Thomas Ryan spoke for the hospitals and brought forcibly to the attention of the Court the peculiar nature of the work which was hardly comparable with the usual lines of industry. The decision of the Court appears to us to be quite fair, but not, according to our knowledge, setting out a scale of remuneration and conditions of work which greatly differs from that in force in many institutions. The question of hours is the great difficulty, and here there is likely to be as much give-and-take as there is at present. This decision coincides with what is possibly the beginning of a wave of unemployment. There is not much difficulty in obtaining unskilled labour in hospitals, for the moment, but we think that this fact will not deter most of the institutions from abiding by the decision of the Industrial Court, although only those who went to arbitration are in any way bound. Many hospitals will be glad to have had a difficult point decided for them.

### TRADE UNIONISTS AND A LONDON HOSPITAL.

ON Sunday morning, July 18, a "grand demonstration and parade" will be held on behalf of the Great Northern Central Hospital. It is an interesting point that the North London Amalgamated Hospital Society is arranging the affair, and that the participators include several important bodies of organised Labour, such as the National Union of Railwaymen, the National Union of Vehicle Workers, the National Coal Porters' Union, the National Union of Corporation Workers, the National Amalgamated Furnishing Trades Association, and the General Workers' Union. Mr. H. J. Dungate, 2 Rhodes Street, Barnsbury, is the Organising Secretary.

### GENERAL LEE'S PORTRAIT AT CARDIFF.

A DISTINGUISHED company was present at King Edward VII. Hospital, Cardiff, to witness the unveiling of the portrait of the late Major-General H. H. Lee, C.B.E., Chairman of the board from

1899 to 1920. Sir William James Thomas performed the ceremony. The artist is Miss Margaret Lindsay Williams. In his speech Sir William said that they would always treasure the portrait which would remind them of General Lee's long and honourable service. The occasion of the ceremony was the annual meeting of the Needlework and Linen Guilds, of which this institution makes more than any other hospital.

### SCHOOL CHILDREN AND WELSH HOSPITALS.

To the scheme lately reported of collections from school children, as well as farmers in Norfolk, must be added an energetic attempt to secure the support of the schools for Carnarvonshire and Anglesey Infirmary. It seems, however, that Mr. W. A. Foster, the hospital's treasurer, is trying for an annual collection. Such an event is usually the subject of correspondence, and, as this correspondence is necessarily formal, its results may be disappointing. We believe the better plan to be that which we have described in The Hospital. This plan being for a perpetual collection, with the money-box opened once a quarter in every school, and a group of children in each school to agree to contribute to it, involves a public meeting of the teachers and the parents to start with. In this way enthusiasm can be aroused, and as the totals collected by each school can be published, a healthy rivalry is created, and a permanent source of income found. Perhaps Mr. Foster can start this in Wales, as it has been started in Norfolk.

### HOSPITALS AND INCREASED SPIRIT DUTIES.

As we anticipated in a previous note, the Government has agreed to continue the rebate granted on rectified spirits of wine used for medicinal preparations in respect of the added duty imposed by this year's budget. An attempt was made by amendments to the Finance Bill to obtain a rebate on all alcohol used at hospitals irrespective of the particular preparations made with it, and to include a rebate on brandy and port wine prescribed by medical practitioners. The Government opposed this amendment on the grounds that it would give a loop-hole to possible misuse. An amendment was also proposed so that a reduction of duty would be made on flavouring essences, eau-de-cologne, etc., but both amendments were defeated. We have on former occasions pointed out the necessity for hospital pharmacists to keep a record of all alcohol used for medicinal preparations, so that the rebate, which is now greatly increased, should be claimed. If any institution is not claiming the rebate, it should take steps to secure this, and so lessen the highly increased bill for drugs.

### A WAR CORRESPONDENT'S APPEAL.

The Royal National Orthopædic Hospital in Great Portland Street has enlisted the sympathy of a powerful friend in Sir Philip Gibbs, the well-known war correspondent, and his graphic style of writing adds weight to the appeal. He tells us that "from all parts of the country the children have come, those little ones who in the old slums of life (better, now, but with a dreadful heritage), dragged themselves on crutches, with a 'dot and carry one,' or lay all their childhood on their backs with pain as their bed-fellow, and no joy of childhood." This hospital, which, it will be remembered, represents several hospitals amalgamated, deals with about 60,000 attendances of out-patients every year, and its 200 beds are nearly always full. That there was a deficit of only £1,400 last year is a sign of energetic management, but the strain of this year's finance is being keenly felt, and we hope that Sir Philip Gibbs' eloquent appeal will touch many hearts.

### MEDICAL M.P. ON HOSPITAL FINANCE.

Colonel Dr. Raw, M.P., is reported to have said that the position of voluntary hospitals is exceedingly serious, not only as regards themselves, but as it affects public health, now and in the future. He has suggested that the Government should appoint a committee to deal with the situation, but this idea has not found favour. He makes another suggestion. It is likely that the passing of the Bill as to health services promised for next session will mitigate the hospital difficulties very largely. In the meantime he urges the Government to provide a special grant from Treasury funds for the hospitals to carry them over the interval. It will be remembered that Sir Arthur Stanley has recently hinted at the possibility of a State grant to clear off debt accumulated through the war in order that they may go forward released of this burden.

### ANOTHER LOCAL HOSPITAL FOR SOUTH WALES.

Lady Glanusk, the wife of the donor, has opened the Ivy Tower War Memorial Hospital, Crickhowell. Lord Glanusk is another believer in the scheme for local hospitals in South Wales, often advocated in The Hospital, and designed Ivy Tower not only as a memorial of the two sons and the brother whom he lost in the war, but as an aid to Brecon Infirmary. The hospital consists of two general wards, two maternity wards, and a clinic, provided with central heating and electric light, and the adaptations have been made by Mr. F. J. Hurley, the honorary architect. The building, once part of Crickhowell Castle, has been previously a school and a private house. The secretary is Mr. John Phillips, and the treasurer Mr. E. Pirie Gordon. Miss M. M. Knott is the matron; a nurse is being appointed, and it is interesting to note that the district nurse and the health visitor reside in the institution. It starts well financially. The sum of £4,000 has been received from grants and donations, and £3,700 has been spent. Annual subscriptions have been promised for £225, and with investments an income of £300 seems assured. A revenue of £500 is required, but Lord Glanusk, the president and chairman, believes that the list of subscribers is far from exhausted.

### EDINBURGH HOSPITAL WEEK.

An elaborate organisation has been started in Edinburgh in the attempt to raise part of the £250,000 needed for the Royal Infirmary there. The number of circulars sent was 300,000, and with the aid of the Edinburgh Food Control Office, it was believed that every householder, indeed every lodger, received one of them. The delivery was made with the help of the Boys' Brigade, Boy Scouts, Church Lads' Brigade, and the Girl Guides. Postmen, we understand, arranged the circulars in batches for easy delivery. The depots in the city numbered 155, and the superintendent and his staff of collectors gave the first three days of the Hospital Week to a canvass, the honesty of which was guaranteed by a book of receipts signed by the superintendent of each depot. There were 3,000 collectors, and a university professor undertook to audit the returns. Beside this general collection, the working-class districts were canvassed to join the Infirmary League of regular subscribers, and a number of matches, fêtes and sports were also held. At the time of writing the total raised is not to hand, but if the number of workers is any guarantee, it should be a large sum.

### TUBERCULOSIS HOSPITAL FOR SOUTHEND.

Southend Corporation Health Committee has approved plans for a tuberculosis hospital, which will contain 106 beds and cost £91,443. The grant from the Ministry of Health, at the rate of £180 per bed, will mean a sum of £19,080, which the Committee does not consider sufficient in view of the increase in the cost of building since last November when the Ministry computed the grant. An increase in the grant is, therefore, being demanded. What is the real present cost per head in a hospital under construction to-day?

### PRIVATE HOSPITALS IN AUSTRALIA.

Australia has private hospitals, and, according to a report from the Chief Health Inspector of Perth, Western Australia, the number of these institutions licensed during the year was ten, and they have been subject to regular inspection. These establishments are said to be in most respects very satisfactory.

### THE BATH HOSPITAL TO MOVE.

It has been decided at a public meeting at Bath to approve the removal of the Royal United Hospital from the city to the site of the War Hospital in Combe Park. Mr. W. Mallett has offered to buy the land for £5,000, and the cost of the removal scheme is expected to be £148,000. The scheme is to begin with a hospital of ninety-eight beds for paying patients, and a maternity block with forty-four beds. How soon this plan will be realised is uncertain.

### HALF-MAST IN HAMPSHIRE!

The chairman of the Royal Hampshire County Hospital has announced the decision of his committee to close "at least two wards to adjust the expense of running the hospital to correspond with the available revenue." The two wards will be closed "as soon as can be fixed, having regard to the hospital's engagement with their staff." This veiled language reads like a threat, or that which is said euphemistically to be a tentative proposal. The response will be interesting, but there is abundant proof that the closing of wards saves an amount of money in ludicrous disproportion to the public benefits thereby withdrawn. A similar step is being taken by the Royal National Hospital for Consumption, in Dublin, the committee of which has decided to reduce the number of beds from 125 to 60, because of an overdraft of £6,600. Mr. E. H. C. Wellesley, the chairman, represents a hospital which receives no Government grant. The institution was opened in 1896 with twenty-four beds, and has subsisted almost wholly on subscriptions, donations, and the weekly sums paid on behalf of, or sometimes by, the patients. Will the patients now being turned away be able to evoke sufficient public sympathy to raise sums necessary to restore the hospitals at full work?

### IRISH WORKMEN AND THEIR HOSPITAL.

The Royal Victoria Hospital, Belfast, has a valuable ally in its Working-Men's Committee, the remarkable record of which was given at the committee's annual excursion to the Throne Hospital on June 19. After Mr. W. Carson, J.P., Chairman of the Working-Men's Committee, had presented a cheque to the treasurer for £360, the latter, Mr. Berrington, recorded that since 1915 the working-men's contribution had risen from £5,076 to £12,500 a year. (In other words, the purchasing power of their contribution has been maintained, notwithstanding the fall in the value of money). The three wards opened in 1914 cost now about £12,000 to maintain, and consequently the working men may be said to support ninety-six beds to the employers' sixty-five. Since the hospital is costing about £55,000 a year, and with an overdraft added, the deficit in the first half-year is over £13,000, an extra £23,000 a year, in fact, is needed. Since the cost of necessities rises the purchasing power of gifts must also be increased. Mr. Berrington therefore asks the working classes and the employers of Belfast to double their contributions. The employees, as the hospital has recorded, have already responded well, and it is possible the total income really will be doubled by a joint effort.

### AMALGAMATION DEFERRED AT BRISTOL.

An important special meeting of the Governors of the Bristol General Hospital was held recently to consider the much-talked-of amalgamation scheme, the rejection of which had been recommended by the General Hospital's Committee. In the end it was decided by forty-two votes to eight to hold a conference between the Amalgamation Committee and the General Hospital's Committee. Amalgamation has been recommended for the sake of economy of effort and finance. It is opposed because of the uncertainty of the Government's proposals and because the effect of amalgamation on individual hospitals is said to be a leap in the dark.

### SALFORD HOSPITAL AND SHOP STEWARDS.

The industrial works in Manchester district are supporting the Royal Salford Hospital's Appeal. In fact, the trade-union shop stewards recommend organised weekly collections. The employers are being asked to arrange the collections, and, we hope, will be asked to contribute so much per head on the plan which has proved so successful at Leeds, and at the several other towns which have since adopted the Employers' Contribution Fund started there.

### AN AUXILIARY TO SUNDERLAND ROYAL INFIRMARY.

The overcrowded state of Sunderland Royal Infirmary and other matters needing improvement has led the Committee to recommend an important change. The Committee proposes to use the present building for emergencies only, and recommends the purchase of 15 to 20 acres of land near by whereon an auxiliary infirmary should be built. Not only are more beds needed, but a pathological department under a salaried chief; a throat, nose, and ear department, and a dental clinic are also desired. Among other comprehensive proposals are two of interest. The first is for a convalescent home where patients can be attended by their own physicians; the second for closer relations with the Eye Infirmary. The Committee thinks that an appeal for these objects would be successful.

### TO REPLENISH THE STOCK OF LINEN.

Miss E. H. Renaut, the matron of the David Lewis Northern Hospital, Liverpool, her nurses, and friends recently organised a sale of work which was the first to be held in the institution since its inception in 1834. The sale of beautiful needlework, both of the fancy and useful variety, displayed on the stalls, in addition to household goods, fruit, flowers, country produce, etc., was held with the object of strengthening the general linen stock of the hospital. The Lady Mayoress of Manchester, who performed the opening ceremony, called attention to the uncomfortable fact that the supply of linen for a large hospital is becoming a very difficult problem. She said that in the old days it was quite an easy matter to collect a large supply of linen, but now, owing to the enormous advance in prices, it is no light task to get even a little. We believe that an appeal for a definite object often succeeds while a request for funds for general purposes fails, because many people like to know exactly upon what their money is to be spent rather than to feel that it is unidentified in the common whirlpool of expenditure.

### INFIRMARY CRITICS OF VOLUNTARY HOSPITALS.

The hospital situation gives additional interest to the annual conference of the Poor-Law Association, held at Hull, which discussed at length the present and future of the Poor-Law infirmary. Thus Mr. J. W. Flint, chairman of the Sheffield Board of Guardians, said that the work of the infirmaries was the most invulnerable part of the Poor-Law, which provided, he said, more than half the hospital accommodation of the country. Lord Astor had announced in the House of Lords that a Bill to reform the Poor Law would be introduced this year. Dr. Addison's Consultative Council was entirely composed of medical experts, and medical men were "notoriously bad men of business." Poor-Law hospitals had become public general hospitals, and only "sentiment" restricted admissions. All restrictions should be removed, and infirmaries should co-operate with local hospitals. Mr. J. Deasy urged a petition to the Minister of Health to allow infirmaries to receive paying patients. But, one may ask, since the well-to-do who occasionally receive treatment invariably also receive a bill according to the means, no drastic alteration of the regulations seems to be required. The waiting-list in voluntary hospitals, Mr. Deasy added, was counter-balanced by the number of vacant beds in the infirmaries, which people refused to enter. The Rev. W. Mahon, of Wakefield, said that, were it not for the Ministry of Health, the infirmaries could relieve the present distress. Guardians should be courageous and do what was right, letting the Ministry do what it dared. Mr. Furness, of Sheffield, believed in free treatment, and was opposed to paying patients. Two resolutions were eventually proposed; the first, in favour of all unnecessary restrictions being removed from the infirmaries, was passed. The second, advocating the free admission of all to them, was rejected.

### MEDICAL PRACTITIONERS IN A POOR-LAW INFIRMARY.

The Wandsworth Board of Guardians has approved a scheme to allow medical practitioners to attend clinical lectures in St. James's Infirmary. Some opposition at first was made, but the Guardians had too much faith in the local practitioners to allow it to defeat the proposal.

### THE RHONDDA HOSPITAL SCHEME.

There appears to be every probability that before long the mining valley of the Rhondda, with its population of approaching a quarter of a million, will be provided with the general hospital which it needs. The scheme which is understood to have been launched successfully is that the colliery companies in the valley shall undertake to find or guarantee the capital required to erect the hospital—namely, £100,000; that the local landowners shall equip it; and that the miners should bear the cost of its maintenance. Four or five colliery companies have already approved of the scheme.

### THIS WEEK'S DRUG MARKET.

Speaking generally, the opinion in drug market circles is fairly prevalent that the prices of many of the articles which have recently receded are not likely to recover; but opinion is divided as to whether the downward tendency will continue much longer, and as to whether we are likely to see any further substantial reductions. In any case, there seems to be no reason why buyers should purchase far ahead of their requirements. Supplies of various articles are coming in more freely from the Continent, and altogether the market is fairly well supplied with most of the commonly used drugs. Citric acid and tartaric acid are both cheaper. The price of menthol has again receded, but there is a somewhat steadier tone in the market for Japanese refined camphor. Lemon oil is again rather lower in value, and the same applies to clove oil and star aniseed oil. Carbolic acid is cheaper. Makers of chloroform have advanced their quotations slightly.

