# Erratum

**Published:** 1920-07-24

**Authors:** 


### Erratum.

ON page 404 of last week's issue, in the paragraph referring to the Sale of Work organised by Miss E. A. Renaut, matron of the David Lewis Northern Hospital, Liverpool, and others, it should have been stated that the opening ceremony was performed by the Lady Mayoress of *Liverpool* (Mrs. Burton W. Eills), not Manchester.

